# Alkane biosynthesis by *Aspergillus carbonarius* ITEM 5010 through heterologous expression of *Synechococcus elongatus* acyl-ACP/CoA reductase and aldehyde deformylating oxygenase genes

**DOI:** 10.1186/s13568-016-0321-x

**Published:** 2017-01-05

**Authors:** Malavika Sinha, István Weyda, Annette Sørensen, Kenneth S. Bruno, Birgitte K. Ahring

**Affiliations:** 1 Bioproducts, Sciences and Engineering Laboratory, Washington State University, 2710 Crimson Way, Richland, WA 99354 USA; 2 Section for Sustainable Biotechnology, Aalborg University Copenhagen, AC Meyers Vaenge 15, 2450 Copenhagen SV, Denmark; 3 Chemical and Biological Process Development Group, Pacific Northwest National Laboratory, Richland, WA 99354 USA

**Keywords:** Advanced biofuels, Alkane biosynthesis, *Aspergillus carbonarius* ITEM 5010, Fungal transformation

## Abstract

In this study we describe the heterologous expression of the recently identified cyanobacterial pathway for long chain alkane biosynthesis, involving the reduction of fatty acyl-ACP to fatty aldehyde and the subsequent conversion of this into alkanes, in the filamentous fungus *Aspergillus carbonarius* ITEM 5010. Genes originating from *Synechococcus elongatus* strain PCC7942, encoding acyl-ACP/CoA reductase and aldehyde deformylating oxygenase enzymes, were successfully expressed in *A. carbonarius*, which lead to the production of pentadecane and heptadecane, alkanes that have not been previously produced by this fungus. Titers of 0.2, 0.5 and 2.7 mg/l pentadecane and 0.8, 1.6 and 10.2 mg/l heptadecane were achieved using glucose, Yeast malt and oatmeal media, respectively. Besides producing alkanes, we found elevated levels of internal free fatty acids and triglycerides in the alkane producing transformant. These findings can indicate that a yet unidentified, native fatty aldehyde dehydrogenase channels back the fatty aldehydes into the fatty acid metabolism, thus competing for substrate with the heterologously expressed fatty aldehyde deformylating oxygenase. These findings will potentially facilitate the future application of robust, fungal cell factories for the production of advanced biofuels from various substrates.

**Electronic supplementary material:**

The online version of this article (doi:10.1186/s13568-016-0321-x) contains supplementary material, which is available to authorized users.

## Introduction

Microbial production of fatty acid derived biofuels, such as fatty acids, fatty alcohols and alkanes has received considerable interest in the past few years as a mean for producing advanced biofuels (or drop-in fuels) which can substitute conventional transportation fuels (Fairley 2011). Among the different advanced biofuels, medium- and long chain alkanes are of high interest due to their similarity in energy content and combustion properties to kerosene and diesel components and their potential to be used directly with existing internal combustion engines running on these fuels (Peralta-Yahya et al. 2012).

**Fig. 1:**
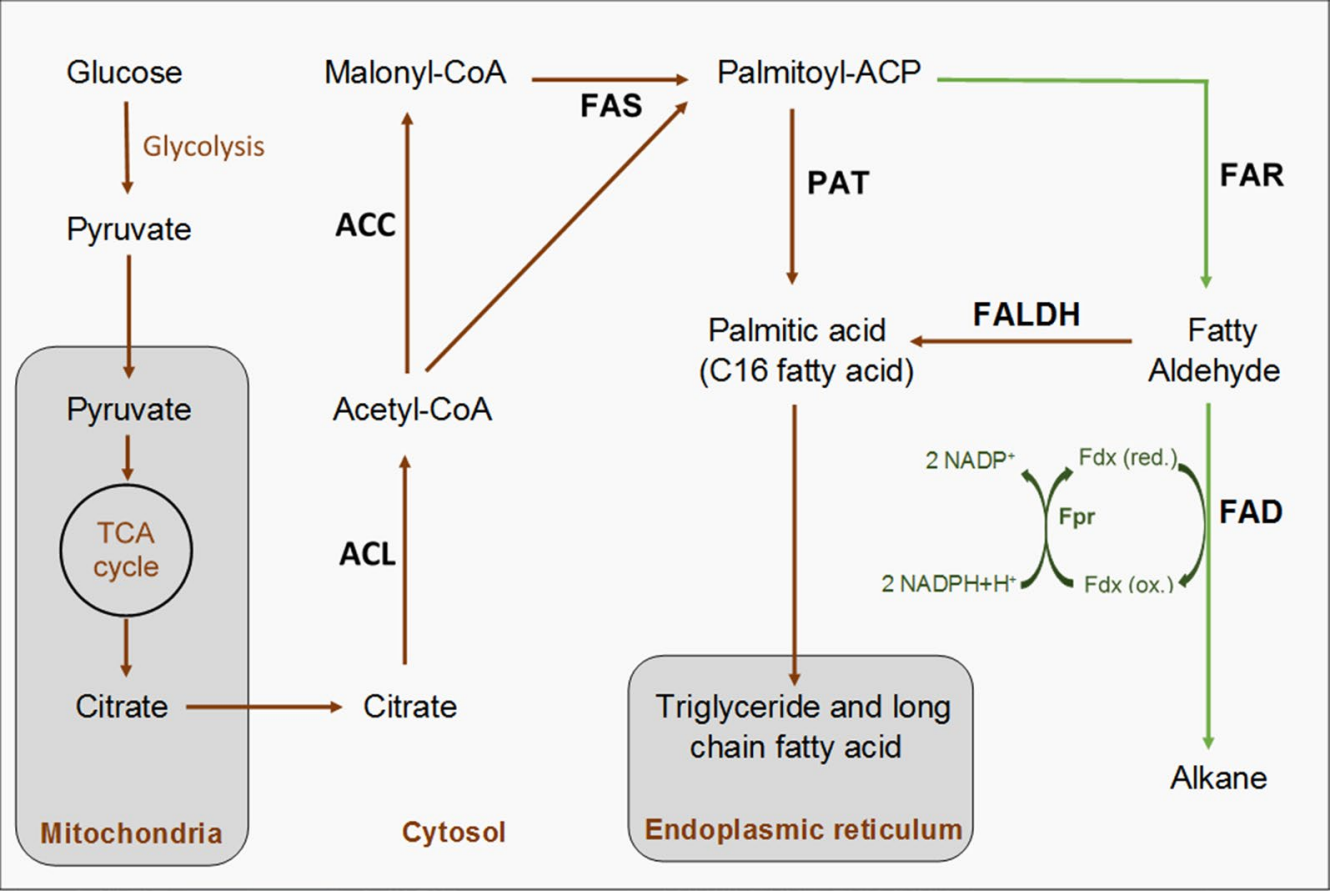
Fatty acid metabolism and heterologous alkane production in fungi. The *S. elongatus* FAR and FADO genes were heterologously expressed in *A. carbonarius*. The introduced alkane biosynthetic pathway is marked with green arrows. The FADO enzyme requires ferredoxin (Fdx), ferredoxin reductase (Fpr) and NADPH as cofactor. The endogenous source supplying these cofactors is not known. The fatty acid metabolism involves the ATP-citrate lyase (ACL), acetyl CoA carboxylase (ACC), fatty acid synthase (FAS) and palmitoyl-ACP thioesterase (PAT). Fatty aldehydes are potentially converted into free fatty acids by the fatty aldehyde dehydrogenase (FALDH)

Production of alkanes by bacteria (Ladygina et al. 2006; Winters et al. 1969) and fungi (Ahamed and Ahring 2010; Sinha et al. 2015; Strobel et al. 2008) has been shown in the past and it is considered to be linked to fatty acid metabolism (Ladygina et al. 2006; Yu et al. 2014) (Fig. 1). However, no studies conclusively described the biosynthesis pathway for the production of these compounds, until recently. Schirmer et al. (2010) identified two genes: acyl-ACP/CoA (FAR) reductase and fatty aldehyde decarbonylase [FADO, recently re-classified to aldehyde deformylating oxygenase (Li et al. 2012)], responsible for the production of C_n_ fatty aldehydes from C_n_ fatty acyl-ACP/CoA and the subsequent conversion of these aldehydes into C_n−1_ alkanes, predominantly pentadecane, heptadecane and methyl-heptadecane, in various cyanobacteria. In this same study, the heterologous expression of these genes in *E. coli* led to alkane titers of 25 mg/l, and based on these findings significant progress has further been made with this microorganism, achieving alkane titers of up to 580 mg/l (Choi and Lee 2013). Most of the research on increasing alkane production was carried out in bacteria, while the use of this pathway is still relatively unexplored in eukaryotic organisms. One study on *Saccharomyces cerevisiae* (Buijs et al. 2015) reported, that the successful expression of FAR and FADO together with the deletion of a fatty aldehyde dehydrogenase (FALDH), encoded by Hfd1, led to the production of long chain alkanes in titers of 22 µg/g dry cells mass. The necessity to disrupt Hfd1 in order to see production of alkanes was in agreement with previous observations made by Kaiser et al. (2013). The Hfd1 enzyme is related to accumulation of fatty acids by degrading fatty aldehydes that would otherwise serve as substrate for the reaction carried out by the FADO enzyme. The production of fatty aldehydes in cells is related to the catabolism of several lipids, such as fatty alcohols, sphingolipids, ether glycerolipids, isoprenoid alcohols, etc. (Rizzo 2014).

Other eukaryotes, such as fungi, are extensively utilized as cell factories for the industrial production of enzymes, organic acids, antibiotics, etc., due to their robustness, tolerance of harsh fermentation conditions and ability to consume a wide range of substrates (Karagiosis and Baker 2012). There have been a number of recent studies focusing on the filamentous fungus *Aspergillus carbonarius* as a potential cell factory for production of different biofuels and biochemicals (Jäger et al. 2001; Sinha et al. 2015; Weyda et al. 2014; Yang et al. 2014, 2015).

In this present study, we utilized cyanobacterial genes in a eukaryotic system for alkane biosynthesis. The work shows that heterologous expression of *Synechococcus elongatus* PCC7942 FAR and FADO (codon optimized for our fungus) in *A. carbonarius* ITEM 5010 led to the *de novo* synthesis of pentadecane and heptadecane, alkanes which have not previously been observed with the parent strain (Sinha et al. 2015).

## Materials and methods

### Fungal strain and growth conditions

*Aspergillus carbonarius* ITEM 5010 (ATCC® MYA-4641™) was used as the parent strain in all the transformations. The strains were grown on Potato Dextrose Agar for 5 days at 30 °C for preparation of conidial suspensions. Sterile double distilled water was added to the plates to collect conidia from the surface of the agar, and the suspension was filtered through sterile Miracloth (Millipore, Billerica MA, USA) and counted in a haemocytometer. Transformants were maintained on minimal medium agar (Samson et al. 2004) supplemented with 100 µg/ml Hygromycin B. Alkane production was studied on glucose (composition same as minimal medium), oatmeal and Yeast Malt medium (YM, 3% yeast extract, 3% malt extract, 10% glucose). Glucose was substituted with oatmeal (20 g/l) for the oatmeal based medium. All chemicals were purchased from Fisher Biotech (Fair lawn, NJ, USA), unless otherwise stated.

### Gene description and codon optimization

*Synechococcus elongatus* strain PCC7942 (ATCC® 33912™) aldehyde deformylating oxygenase (Synpcc7942_1593) and acyl-ACP reductase (Synpcc7942_1594) were the two genes used in this study. Codon optimization and gene synthesis were carried out by Genscript USA Inc. (Piscataway, NJ, USA). The optimization was carried out based on the codon usage frequency of the closely related strain *Aspergillus niger* (Abarca et al. 2004). The sequence of the codon optimized FAR and FADO genes can be retrieved with Genbank accession numbers KX903286 and KX903287, respectively.

### Plasmids and expression vectors

The promoters of genes encoding *Tef1* and *CoxA*, from *Aspergilus nidulans* and *A. niger*, respectively, and the terminators of genes *TrpC* and *CoxA* also originating from *A. nidulans* and *A. niger*, respectively, were used for the heterologous expression of the codon optimized genes in *A. carbonarius*. The promoters and terminators were PCR amplified from the genomic DNA of their parent strains (kindly donated by Pacific Northwest National Laboratory, Richland, WA). The sequences of oligonucleotides used in this study are provided in Additional file 1: Table S1.

The FAR/FADO expression vector consisted of the codon optimized FAR flanked by *Tef1* promoter and *CoxA* terminator and the codon optimized FADO with *CoxA* promoter and *TrpC* terminator. The promoters, genes and terminators were cloned into plasmid *pCB1004* (Fungal Genetics Stock Center, Manhattan, KS, USA), which contains Hygromycin B fungal selection marker, by Gibson Assembly Cloning (New England Biolabs, Ipswich, MA, USA), according to the manufacturer’s protocol. The resulting expression vector, *pCB1004TDR* (Additional file 1: Figure S1), was used to transform protoplasts of the parent strain.

### Transformation of *A. carbonarius* ITEM5010

Ectopic integration of the expression vector into the genome of *A. carbonarius* ITEM 5010 was achieved via protoplast transformation. Protoplast preparation and transformation was carried out as previously described by Gallo et al. (2014). The transformant heterologously expressing the FAR and FADO genes is referred to as TDR transformant.

### Selection of transformants, DNA extraction, and expression analysis

Randomly selected transformants were isolated on selective minimal medium agar. Genetically stable homokaryons were achieved by three successive rounds of plating conidia of the selected transformants on selective medium and isolation of single colonies. Genomic DNA (gDNA) of transformants was isolated by the Cetyl trimethylammonium bromide (CTAB) and phenol–chloroform extraction method, previously described by Lee et al. (1988) and was used as template for the PCR verification of the transformants for the presence of the correct inserts, using oligonucleotides IW501 and IW512 (Additional file 1: Table S1) and Phusion polymerase (New England Biolabs, Ipswich, MA, USA), according to the manufacturer’s protocol. Based on the PCR verification, seven transformants were selected for further analysis for expression of the inserted genes. Total RNA was extracted by following the manufacturer’s protocol of RNeasy Plant Mini Kit (Qiagen, Valencia, CA, USA). Complementary DNA (cDNA) was synthesized from mRNA by using the Verso cDNA Synthesis Kit (Thermo Scientific, Pittsburgh, PA, USA), according to the manufacturer’s protocol and verified by PCR using oligonucleotides presented in Additional file 1: Table S1. The PCR verification consisted of the amplification of a few hundred base pairs of the terminal region of both the FAR and FADO genes from the selected transformants. Beta-actin was used as control to confirm that cDNA had been synthesized and that the samples did not contain traces of genomic DNA that would interfere with proper assessment of the PCR results. Amplification of a short terminal region of beta-actin yields fragments of different sizes from gDNA and cDNA due to the presence of an intron.

### Southern blot analysis

The hybridization probe for the southern blot analysis of the TDR transformants was designed to consist of a single fragment containing FADO, *TprC* terminator, *tef1* promoter and FAR sequence, originating from the previously constructed expression vector (Fig. 3a). The DNA probe was biotin labeled by using Pierce North2South Biotin Random Prime Kit (Thermo Fisher Scientific, Rockford, IL, USA) according the manufacturer’s protocol. Genomic DNA of the transformants was extracted as described above and digested with restriction enzymes BamHI, PstI, and NdeI (all purchased from Thermo Scientific, Rockford, IL, USA). Southern blotting was carried out using Whatman Turboblotter transfer system (GE Healthcare Life Sciences, Pittsburg, PA, USA) and the nucleic acid detection was carried out by using Pierce Chemiluminescent Nucleic Acid Detection Module Kit (Thermo Fisher Scientific, Rockford, IL, USA), both according to the manufacturer’s protocol.

### Fermentation conditions

Flask fermentation of the selected transformant and the parent strain were carried out using three different carbon based media: glucose, oatmeal, and YM medium. All three media were supplemented with 1% Tween 80. Each flask (500 ml, non-baffled) contained 100 ml medium which was inoculated with fungal conidia to a final concentration of 5 × 10^5^ conidia/ml. The cultures were grown on 30 °C at 140 rpm for 6 days in a shaking incubator. All fermentations were carried out in triplicates, and a control flask was run parallel without any fungal inoculum.

### Internal free fatty acid and triglyceride assay

Lyophilized hyphae of the enhanced expression strain and the parent strain, cultured on the glucose medium, were prepared for analysis using a method previously described by Tamano et al. (2013). The analysis for free fatty acid and triglyceride concentrations was carried out using a commercial free fatty acid kit (Free fatty acids, Half-micro test kit; Roche Applied Science, Mannheim, Germany) and a triglyceride kit (Triglyceride colorimetric assay kit; Cayman Chemical Company, Ann Arbor, MI, USA) following the manufacturer’s protocol.

### Fatty acid methyl ester (FAMEs) analysis

Fatty acid methyl ester reaction of the samples was carried out following a method developed by O’Fallon et al. (2007) with lyophilized hyphae (here and below DCW) of the TDR transformant and the parent strain, originating from the culturing on glucose medium, using tridecanoic acid as internal standard. The FAMEs were detected by using gas chromatography (GC)/Flame ionization detector (FID) GC-system model # 6890 N (G1540 N), Agilent Technologies, Wilmington, DE, USA, equipped with a DB-WAX column (30 m × 0.53 mm × 1.00 µm, Agilent Technologies, Wilmington, DE, USA). Retention times of the detected peaks were compared to authentic standards (FAME mix C8-C22, Sigma-Aldrich, St. Louis, MO, USA).

### GC/MS sample preparation and alkane analysis

After fermentation, the cultures were filtered through Miracloth (Millipore, Billerica MA, USA). Twenty milliliters of each filtrate was transferred to 50 ml glass centrifuge tubes. Ten milliliters of hexane was added to all tubes, followed by ultra-sonication for 60 min at room temperature, and then vortex for 5 min at maximum speed. Finally, the tubes were centrifuged at 2800*g* for 10 min and the upper hexane layer was analyzed by gas chromatography/mass spectrophotometry (GC/MS) (7890A GC-system with 5975C inert XL E1/C1 MSD model # G3174A, Agilent Technologies, Wilmington, DE, USA). The samples were analyzed on DB-5MS, non-polar (30 m × 0.250 μm × 0.25 × μm) column, using the following method: 1 μl splitless injection (inlet temperature held at 300 °C) onto the column, the oven was held at 30 °C for 1 min. The temperature was ramped up to 200 °C by 10 °C/min and was held at 200 °C for an additional 1 min. The flow rate of the carrier gas helium was 20 ml/min. Retention times of product peaks were compared with authentic standards (Sigma Aldrich, St. Louis, MO, USA) to confirm peak identity. The quantification of the compounds was done by the external standard method (Zhang et al. 2007, 2008). Three commercial alkane standards (Sigma Aldrich, St. Louis, MO, USA) were prepared at 0.5, 1.0, 1.5, 2.0 and 2.5 mg/l (ppm) concentrations in hexane. Pentadecane and heptadecane standards were used to quantify the alkanes. Tridecane standard was used as internal standard. For accurate quantification purposes, undiluted and 10 times diluted samples were run.

## Results

### Selection of correct transformants and southern blot analysis

**Fig. 2:**
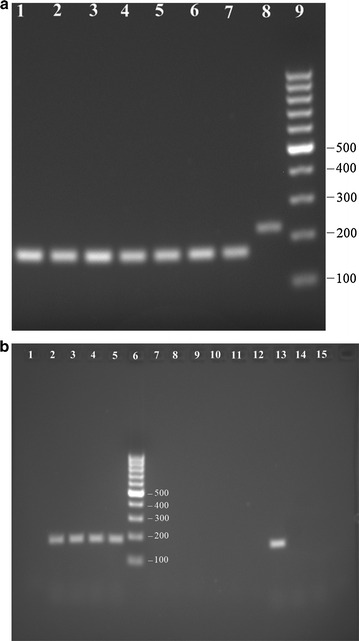
Results of cDNA analysis. **a** PCR with the actin primers on the cDNA of transformants 1–7 (*lanes* 1–7) and on the gDNA of the parent strain (*lane* 8); 100 bp DNA ladder on *lane* 9 (size in base pairs). **b** PCR with FADO primers on the cDNA of transformants 1–7 (*lanes* 1–5, 7 and 8) and with the FAR primers (*lanes* 9–15); 100 bp DNA ladder on *lane* 6 (size in base pairs)

The transformants were verified by PCR to determine if integration of the vector construct had occurred, followed by analysis of expression from cDNA (Fig. 2).The quality of the synthesized cDNA of the transformants was verified by amplifying a short terminal fragment of the beta- actin gene as control. A fragment of ~160 base pairs and ~220 base pairs represent cDNA and gDNA, respectively (Fig. 2a). Only one of seven potential TDR transformants (transformant 5) showed expression of both genes (Fig. 2b), which was used for further analysis for alkane production. Transformants not expressing both FAR and FADO were not analyzed further for alkane production.

**Fig. 3:**
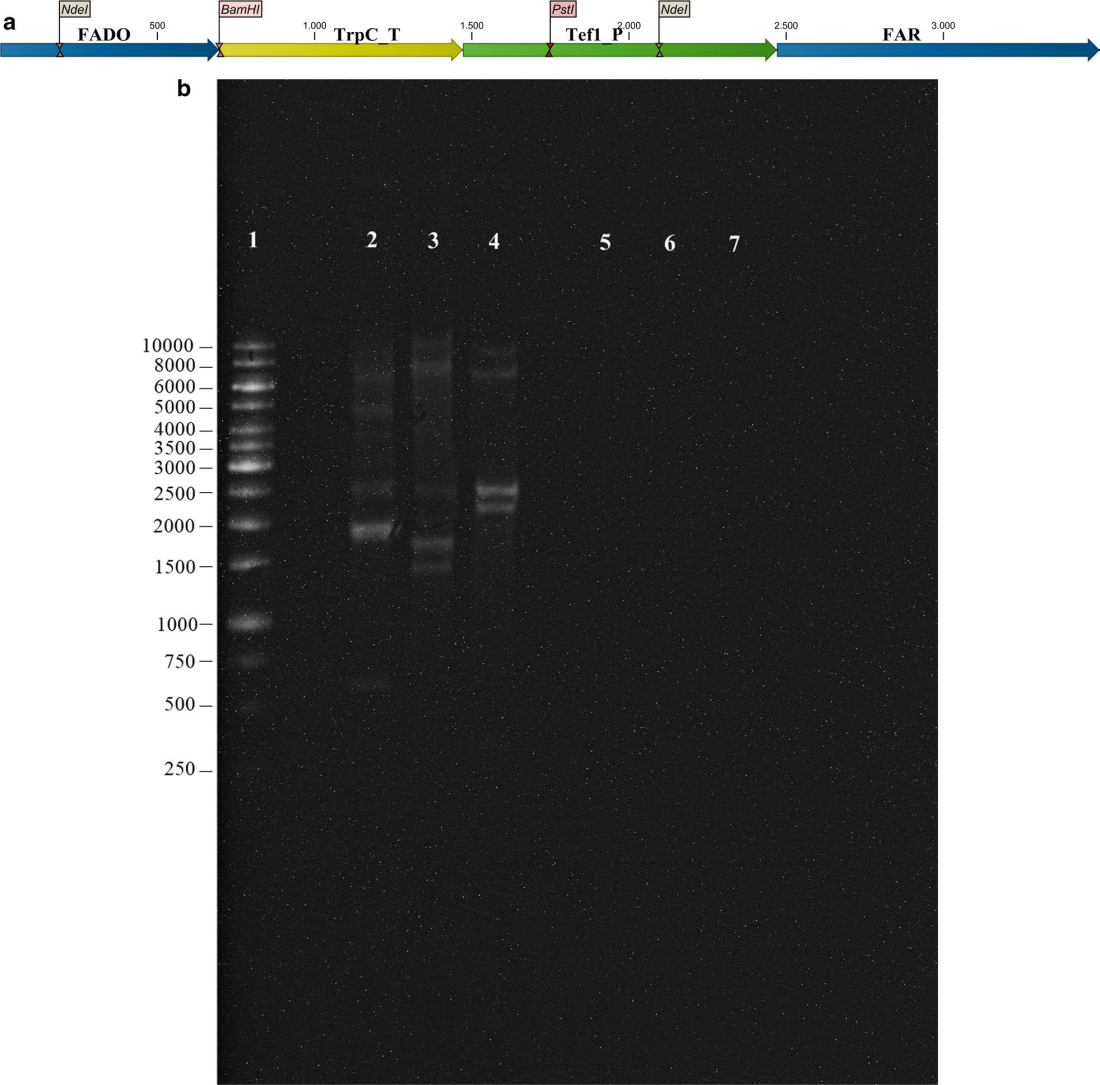
FAR/FADO gene copy number determination in the selected TDR transformant. **a** Hybridization probe used for the Southern blot analysis; FADO fatty aldehyde deformylating oxygenase; TrpC_T terminator region of *TrpC*; Tef1_P promoter region of *Tef1*; FAR fatty acyl-ACP/CoA reductase; BamHI, NdeI, PstI restriction enzyme cut sites. **b** Southern blot analysis. *Lane* 1 contains 1 kb DNA ladder (size in base pairs); *lanes* 2–4 shows the hybridization of the probe to the BamHI, PstI and NdeI digested gDNA of the TDR transformant; *lanes* 5–7 shows the hybridization of the probe to the BamHI, PstI and NdeI digested gDNA of the parent strain

Protoplast mediated transformation generally results in multiple random insertions of the introduced DNA in the genome, therefore gene copy number of the TDR transformant was determined by southern blot analysis (Fig. 3b). The analysis revealed that at least three copies of the expression vector consisting of FAD and FADO genes are present in the genome of the TDR transformant. No hybridization of the probe occurred with the digested gDNA of the parent strain.

### Alkane production

**Fig. 4:**
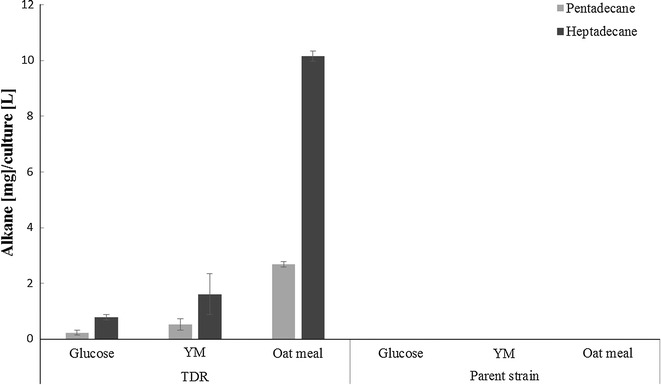
Pentadecane and heptadecane production of the TDR transformant on glucose, YM and oatmeal medium, after 5 days of incubation. Amounts are presented as mg alkane per liter culture. No pentadecane or heptadecane was detected with the parent strain

The alkanes produced by the transformants and the parent strain were analyzed by GC/MS. Pentadecane and heptadecane were produced by the TDR transformant, but were not observed with the parent strain. The amount of these two alkanes produced varied with the different carbon sources (Fig. 4). Fermentation samples were analyzed on day 2, 3, 4, 5 and 6. The maximum amount of alkanes was produced on day 5, and no alkanes were observed until after day 3. Out of the three media, the oatmeal based medium generated the highest amount of these alkanes. The amounts of pentadecane produced from glucose, YM and oatmeal media were 0.2, 0.5 and 2.7 mg/l, respectively. Meanwhile, the heptadecane titers from glucose, YM and oatmeal based media were 0.8, 1.6 and 10.2 mg/l, respectively.

### Internal free fatty acid, FAMEs, and triglyceride production

**Fig. 5:**
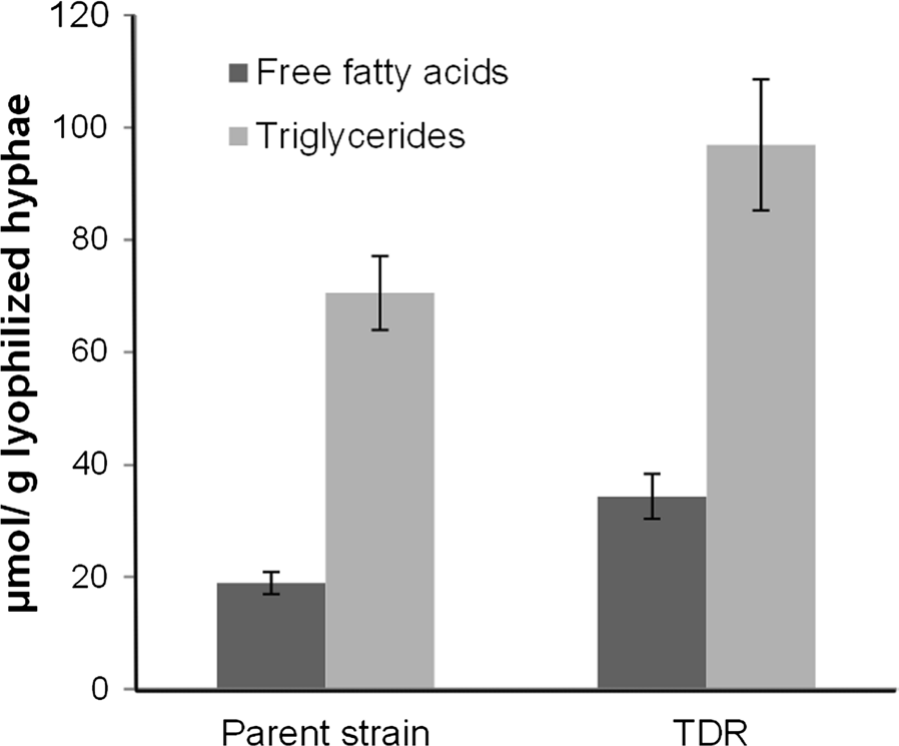
Results of the internal free fatty acid and triglyceride assays. Amounts are presented as µmol free fatty acid or triglyceride per gram of lyophilized hyphae. TDR marks the transformant expressing FAR and FADO. The *graph* shows the averages and standard deviations of triplicate samples originating from the glucose medium

The TDR transformant showed higher levels of internal free fatty acids and triglycerides, compared with the parent strain. The assays show 18.9 and 34.4 µmol free fatty acids and 70.6 and 96.9 µmol triglycerides per gram of lyophilized hyphae for the parent and TDR strains, respectively (Fig. 5). The strains were only analyzed on the glucose medium.

During the FAMEs analysis, four different fatty acids were detected in both the TDR transformant and the parent strain grown on glucose medium: palmitic acid (C16:0), stearic acid (C18:0), oleic acid (C18:1), linoleic acid (C18:2). Relative to the parent strain, the quantity of each of these fatty acids were slightly higher in the TDR transformant (Table 1).

**Table 1:** Results of the FAMEs analysis

Strain	Palmitic acid (C16:0), mg/g DCW	Stearic acid (C18:0), mg/g DCW	Oleic acid (C18:1), mg/g DCW	Linoleic acid (C18:2), mg/g DCW
Parent strain	40.94 (±1.26)	19.72 (±0.03)	68.71 (±3.14)	51.02 (±1.58)
TDR	47.78 (±2.76)	22.34 (±2.77)	79.97 (±5.42)	65.40 (±5.45)

Data derived from triplicate samples

## Discussion

The heterologous expression of the *S. elongatus* PCC7942 codon optimized FAR and FADO in *A. carbonarius* ITEM 5010 resulted in the production of alkanes, specifically pentadecane and heptadecane, which have not previously been seen produced by this fungus (Sinha et al. 2015). These results concur with the findings of Schirmer et al. (2010), where the FAR and FADO were tied to the production of mostly these two alkanes in cyanobacteria. Therefore, we assume that in the TDR transformant, the production of pentadecane and heptadecane follows a similar pathway to the one described in cyanobacteria, where FAR reduces C_n_ fatty acyl-ACP to C_n_ fatty aldehyde, which is further converted to C_n−1_ alkane by FADO. Based on this, the production of C15:0 and C17:0 alkanes such as pentadecane and heptadecane would require C16:0 (palmitic acid) and C18:0 (stearic acid) fatty acid intermediates, which matches the fatty acid profile of *A. carbonarius* as shown by the FAMEs results. The proportion of the detected fatty acids is shown by the FAMEs analysis, indicating that oleic and linoleic acid are more abundant in the cells compared with palmitic and stearic acid in both the TDR and the parent strain. This corresponds with the fatty acid composition observed in other species of *Aspergillus* (Fraga et al. 2008).

Further, the heterologous expression of FAR and FADO genes in *E. coli* produced a mixture of primary alcohol and 1-aldehyde along with pentadecane and heptadecane (Schirmer et al. 2010), however, these alcohol and aldehyde intermediates were not detectable in the analysis of our TDR transformant.

Previous observations made by Buijs et al. (2015) regarding the existence of competing pathways for the fatty aldehyde substrate, generated by the heterologously expressed FAR enzyme from fatty acyl-ACP, support our results on elevated free fatty acid and triglyceride content of the TDR transformant. The increase in fatty acids is assumed to be attributed to an endogenous FALDH, which channels the fatty aldehyde efficiently back into the fatty acid metabolism; in this way competing with the heterologously expressed FADO and alkane production. However, the FAMEs analysis shows a higher degree of increase of oleic and linoleic acids compared with palmitic and stearic acids, which may be attributed to the fatty acids which were channeled back by the FALDH and were further directed into the fatty acid metabolism. Even though no such FALDH has yet been identified and described in filamentous fungi, it is considered as a “housekeeping” enzyme and it has been identified in cyanobacteria (Kaiser et al. 2013), yeast (Buijs et al. 2015; Iwama et al. 2014) and human cells (De Laurenzi et al. 1996).

The activity of the FADO was shown to require ferredoxin (F), ferredoxin (FNR) reductase, and NADPH as cofactor (Schirmer et al. 2010). In a *S. cerevisiae* strain heterologously expressing FAR and FADO, no alkane production was observed without the co-expression of a bacterial F and FNR, even though homologs to these were already present in the microorganism. This was attributed to the fact that these native proteins were located in the mitochondria, thus being inaccessible for the cytosolic alkane pathway. Interestingly, in the TDR transformant this phenomenon was not observed as it was able to produce pentadecane and heptadecane without the additional heterologous expression of an F/FNR system, therefore a system for supplying cofactors to FADO in the cytosol must already exist in the fungus.

In this study we have introduced the prokaryotic alkane biosynthesis pathway from *S. elongatus* PCC7942 in a eukaryotic system, more specifically in the filamentous fungi *A. carbonarius* ITEM 5010. This fungus, which is already a known producer of important jet fuel components such as tetradecane and hexadecane is also able to metabolize a variety of different carbon sources, including lignocellulosic biomasses. Our further addition of the biosynthesis pathway for pentadecane and heptadecane production in this fungus sets the stage for the production of a more complete mixture of future infrastructure ready drop-in advanced biofuels. However, as the application of *A. carbonarius* can be tightly regulated in certain countries due to its potential ability to cause aspergillosis, we recommend that the required safety standards are followed when working with this fungus.

## Additional file

**Additional file 1.** Additional table and figure.

## Abbreviations

FFA: free fatty acid
TG: triglyceride
FAME: fatty acid methyl ester
YM: yeast malt
