# Supplementary material for: Alkane biosynthesis by Aspergillus carbonarius ITEM 5010 through heterologous expression of Synechococcus elongatus acyl-ACP/CoA reductase and aldehyde deformylating oxygenase genes
Source: AMB Express. 2017 Jan 5;7:18. doi: 10.1186/s13568-016-0321-x (PMC5216010; doi:10.1186/s13568-016-0321-x)
Supplement: Supplementary file 1 — Additional file 1. Additional table and figure. [file 13568_2016_321_MOESM1_ESM.pdf]

Supplementary material

Title: Alkane biosynthesis by *Aspergillus carbonarius* ITEM 5010 through heterologous expression of *Synechococcus elongatus* acyl-ACP/CoA reductase and aldehyde deformylating oxygenase genes

Journal: AMB Express

Authors: Malavika Sinha[1¶], István Weyda[2¶], Annette Sørensen[1,2], Kenneth S. Bruno[3], and Birgitte K. Ahring[1*]

Affiliation:

[1]Bioproducts, Sciences and Engineering Laboratory, Washington State University, 2710 Crimson Way, Richland, WA 99354, USA.

[2]Section for Sustainable Biotechnology, Aalborg University Copenhagen, AC Meyers Vaenge 15, DK-2450 Copenhagen SV, Denmark.

[3] Chemical and Biological Process Development Group, Pacific Northwest National Laboratory, Richland, WA 99354, USA

*** Corresponding author**: Birgitte K. Ahring (bka@wsu.edu)

[¶] M. Sinha and I. Weyda and contributed equally to this work

Supplementary Table S1. Oligonucleotides used in this study

Name	Sequence	Annotation
IW501	GGATCCACTAGTTCTAGAGCGG CCGCATGCCTGATCCAGCCCCC	CoxA promoter Forward
IW502	ACTGAGGCATTGTCCTGGTGGG TGGGTTG	CoxA promoter Reverse
IW503	CACCAGGACAATGCCTCAGTTG GAAGCC	Aldehyde deformylating oxygenase Forward
IW504	CGTTAAGTGGATCCTAGACAGC GGCCAAACC	Aldehyde deformylating oxygenase Reverse
IW505	GGCCGCTGTCTAGGATCCACTT AACGTTACTGAAATC	TrpC terminator Forward
IW506	CTGGATCAGGCATTCGAGTGGA GATGTGGAG	TrpC terminator Reverse
IW507	CATCTCCACTCGAATGCCTGAT CCAGCCCCC	Tef1 promoter Forward
IW508	TCAACCCAAACATTGTCCTGGT GGGTGGGTTG	Tef1 promoter Reverse
IW509	ACCCACCAGGACAATGTTTGGG TTGATTGGG	Acyl-ACP/CoA reductase Forward

IW510	AGAATCTGACACCCTAAATGGC GAGAGCCAG	Acyl-ACP/CoA reductase Reverse
IW511	TCTCGCCATTTAGGGTGTCAGA TTCTGTACAAG	CoxA terminator Forward
IW512	AAAGCTGGAGCTCCACCGCGGT GGCTCGCTTGATCAGCTCACTT C	CoxA terminator Reverse
IWADf	AAGCCAACCGCCAAAATTTG	FADO gene, cDNA analysis
IWADr	AGACAGCGGCCAAACCATAGG	
IWARf	ATGAGCGCTGCTGAGATGGC	FAR gene, cDNA analysis
IWARr	GAGGTTGGAAGCCATGCCGCA	
Actin F	AGAGCGGTGGTATCCATGAG	Actin gene, used in cDNA analysis
Actin R	TGGAAGAGGGAGCAAGAGCG	

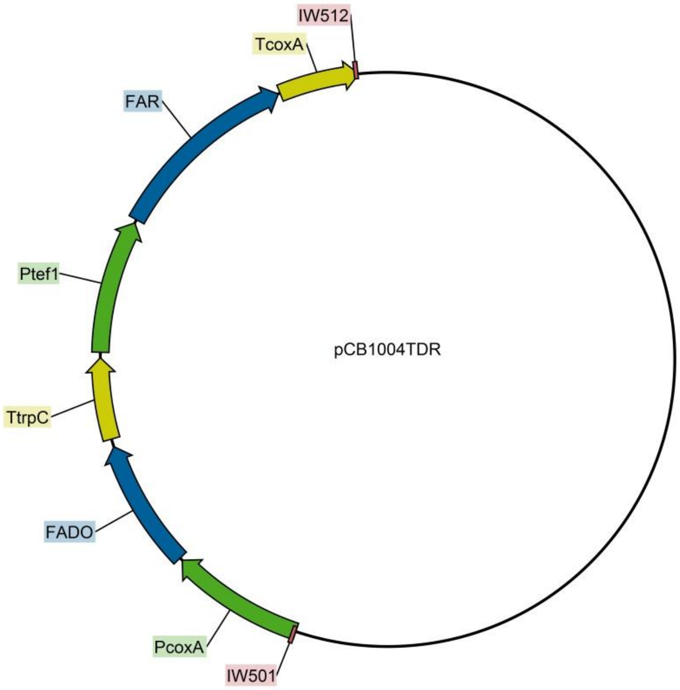

Supplementary Figure S1 Plasmid map of *pCB1004TDR* expression vector. PcoxA Promoter region of *CoxA*; FADO fatty aldehyde deformylating oxygenase; TtrpC Termniator region of *TrpC*; Ptef1 Promoter region of *Tef1*; FAR fatty acyl-ACP/CoA reductase; TcoxA Terminator region of *CoxA*; IW501 Forward primer annealing to PcoxA; IW512 Reverse primer annealing to TcoxA. IW501 and IW512 were used for the PCR verification of transformants for correct inserts
